# The association between acromial anatomy and articular-sided partial thickness of rotator cuff tears

**DOI:** 10.1186/s12891-021-04639-1

**Published:** 2021-09-06

**Authors:** Cen Tao Liu, Jia Qing Miao, Hua Wang, Heng an Ge, Xian Hui Wang, Biao Cheng

**Affiliations:** 1 Department of Orthopedics, Putuo People’s Hospital, Tongji University, No. 1291 Jiangning Road, Putuo District, 200060 Shanghai, China; 2 Department of Orthopedics, Shanghai Tenth People’s Hospital, Tongji University School of Medicine, No. 301 Yanchang Middle Road, Jing’an District, 200072 Shanghai, China

**Keywords:** Articular-sided partial thickness of rotator cuff tears, Acromial anatomy, Acromion index, Critical shoulder angle

## Abstract

**Background:**

Acromial anatomy has been found to be correlated with degenerative full-thickness rotator cuff tears in current studies. However, research on the relationship between acromial anatomy and articular-sided partial thickness of rotator cuff tears (PTRCTs) is still lacking. The purpose of this study was to evaluate whether these imaging graphic parameters exhibit any association between acromial anatomy and degenerative articular-sided PTRCTs.

**Methods:**

Between January 2016 and December 2018, a total of 91 patients without a history of trauma underwent arthroscopy as an articular-sided PTRCT group. In the control group, with age- and sex-matched patients, we selected 91 consecutive outpatient patients who underwent shoulder magnetic resonance imaging (MRI) because of shoulder pain and an MRI diagnosis of only synovial hyperplasia and effusion. MRI was used to measure the acromial type, acromiohumeral distance (AHD), lateral acromial angle (LAA), acromion index (AI), and critical shoulder angle (CSA) by 2 independent observers.

**Results:**

The acromion type, AHD and LAA showed no difference between degenerative articular-sided PTRCTs and controls (*P* = 0.532, 0.277, and 0.108, respectively). AI and CSA were significantly higher in degenerative articular-sided PTRCTs (*P* = 0.002 and 0.003, respectively). A good correlation was found between AI and CSA to measurement(Pearson correlation coefficient = 0.631).

**Conclusions:**

Our study revealed that higher AI and CSA were found in degenerative articular-sided PTRCTs. Acromial anatomy with a large acromial extension was associated with the occurrence of degenerative articular-sided PTRCTs.

## Background

Rotator cuff tears (RCTs) are a common shoulder disease and are highly prevalent among elderly people [1]. In addition to age, dominant arm and systemic diseases, a number of studies have reported that the risk factors for RCTs are related to the anatomic variations of acromions [2]. Regarding articular-sided partial thickness rotator cuff tears (PTRCTs) as the maximum proportion of PTRCTs [3], age-related degeneration has been reported in many articles [4]. Whether there is an association between acromial anatomy and degenerative articular-sided PTRCTs is still unclear.

Bigliani et al. [5] found three different types of acromions in 1991, and the one most commonly associated with RCT was the hook type. Other articles reported an acromion type without a relationship with RCTs [6]. Although rarely found in RCTs, Natsis et al. [7] reported convex acromions except for the three types noted above. In addition to the type of acromion, Banas et al. [8] reported a low lateral acromial angle (LAA) associated with RCT, which was later confirmed by other authors [9]. In full thickness rotator cuff tears (FTRCTs), Nyffeler et al. measured the index of the acromion and found that a large lateral extension of the acromion may exist [10]. Goutallier et al. [11] reported that the acromiohumeral distance (AHD) is smaller in RCTs, while others reported a decrease in AHD after FTRCTs [12]. Recently, some authors focused on the critical shoulder angle (CSA), as proposed by Moor et al. [13], and showed a strong correlation with RCTs and osteoarthritis.

The parameters described above were measured singly or were focused on the full thickness of the RCT. The results are still controversial. Humeral head translation secondary to FTRCT has been reported [12], while in shoulder model research, CSA and AI were positively correlated with humeral head inferior-superior translation [14], which may be biased in regarding whether it is the cause or the effect of FTRCTs. Studies have shown that osteophyte under acromion associated with bursal sided PTRCTs [15], while tears on the articular side seem to be more common than those on the bursa side, the undersurface of the acromion usually was intact in articular-sided PTRCTs [16]. Therefore, this study mainly focused on the articular-sided PTRCTs. While Yoo et al. [4] found a higher CSA in articular-sided PTRCTs, other acromion parameters were not described. In this article, we evaluated the relationship of these common parameters (acromial type, acromiohumeral distance, lateral acromial angle, acromion index, and critical shoulder angle) with articular-sided PTRCTs.

## Methods

### Patients

We retrospectively evaluated data from consecutive patients at our institution from January 2016 until December 2018 who underwent arthroscopically confirmed isolated articular-sided PTRCTs. The patients we included had grade III articular-sided PTRCTs (exceeding 50 % of the thickness of the tendon [17], was the failure predictors after conservative treatment of symptomatic PTRCT [18]); the diagnosis was based on their symptoms (pain or shoulder dysfunction), physical examination, and magnetic resonance imaging (MRI) and was finally confirmed by arthroscopy. These patients had supraspinatus or infraspinatus tear isolated or combined, and no subscapular was involved. All patients experienced failed conservative treatment for at least 3 months preoperatively, including activity modification, nonsteroidal anti-inflammatory drugs, physiotherapy, and steroid injections. A total of 91 patients (group 1) had shoulder joint sagittal and coronal oblique MRI before the surgery. The control group (group 2) contained 91 outpatient patients who underwent shoulder MRI and were age- and sex-matched. All of these patients underwent MRI because of shoulder pain, and were diagnosed (based on the MRI) with only synovial hyperplasia and effusion. The exclusion criteria were as follows: (1) articular-sided PTRCTs caused by trauma; (2) prior surgery on the same shoulder; (3) scapula or greater tuberosity fractures; (4) glenohumeral arthritis; (5) combined with other lesions (SLAP or long head biceps lesions); (6) shoulder infections; (7) other types of PTRCTs or FTRCTs; and (8) insufficient imaging evaluation.

### Measurements

MRI is largely comparable to CT scan for evaluation of the glenoid in less extreme Walch glenoids [19] and accuracy in acromial morphology measurement [20]. MRI examinations were accomplished using a 1.5-T unit (Intera-Power, Philips Medical Systems, Best, the Netherlands). Each patient were placed in the supine position with their arms abducted to the sides. The elbows were extended and the palm upward. T1-weighted imaging, T2-weighted imaging and T2-weighted imaging with fat suppression in Sagittal, coronal oblique and axial planes were performed. The slice thickness was 5 mm with an interslice gap of 1 mm. All MRIs were performed by the same facility. T1-weighted imaging were used to categorized the acromial type and made the measurement of LAA, AI, AHD and CSA.

The classification of acromial type was described by Bigliani et al. [5] and Natsis et al. [7]. We measured the undersurface of the acromion on sagittal oblique shoulder MRI and categorized the patients into four types: type I flat, type II curved, type III hooked and type IV convex.

**Fig. 1:**
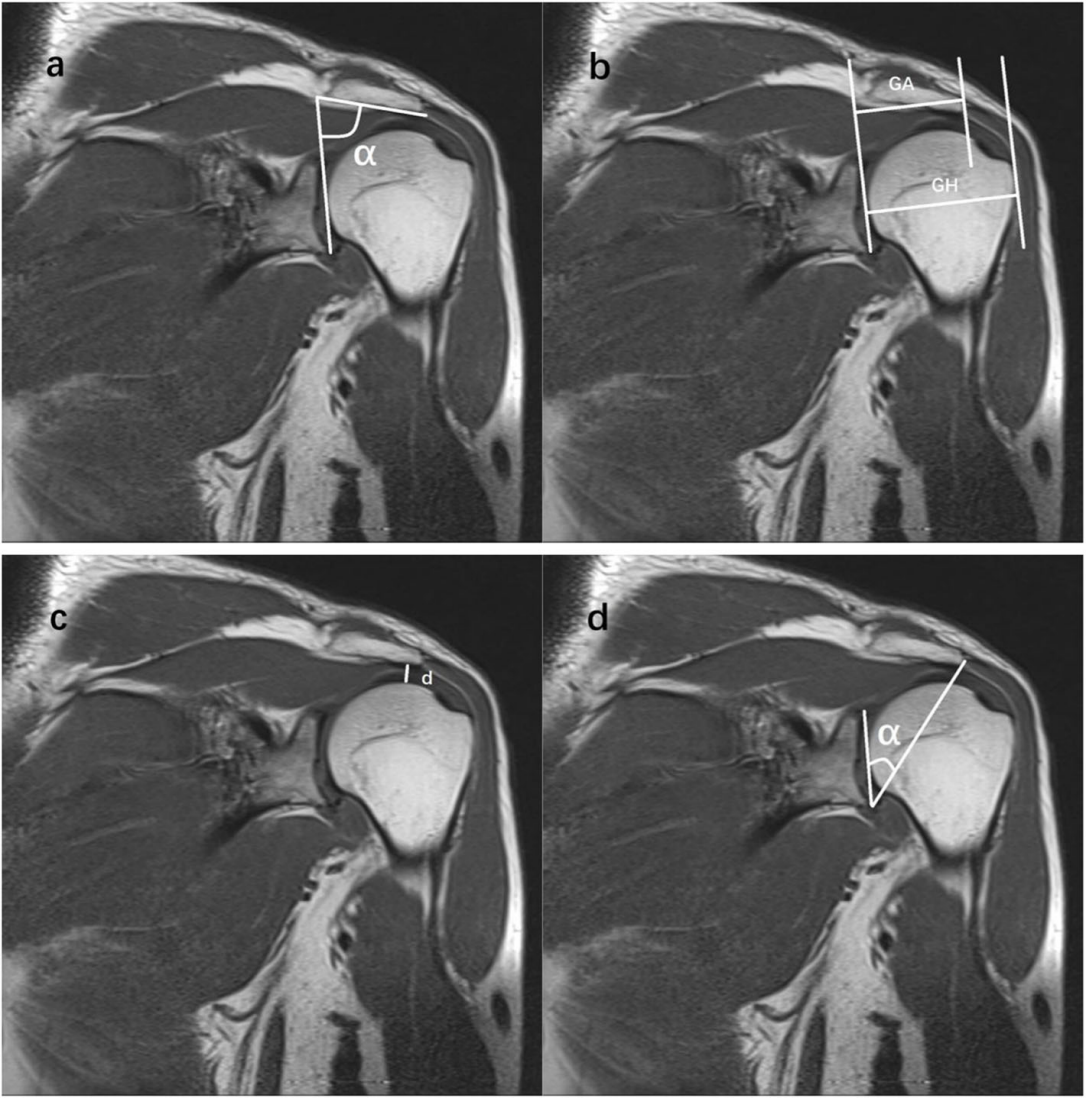
Measurement parameters of acromial morphology. **a** lateral acromial angle (LAA, α), **b** acromion index (AI, AI = GA/GH), **c** acromiohumeral distance (AHD, d), **d** critical shoulder angle (CSA, α)

The LAA was measured according to Banas et al. [8] on the coronal oblique shoulder MRI, and the angle was formed by a line parallel to the acromion undersurface and another line drawn along the superior and inferior most lateral glenoid (Fig. 1a).

AI measured the proportion of the distance from the glenoid plane to the most lateral point of the acromion and the distance from the glenoid plane to the most lateral point of the humeral head on coronal oblique shoulder MRI as described by Nyffeler et al. [10] (Fig. 1b).

The measurement of AHD according to Goupille et al. [21] on the coronal oblique shoulder MRI revealed the shortest distance between the inferior plane of the acromion and the superior plane of the humeral head (Fig. 1c).

We measured the CSA according to the method described by Moor et al. [13] on the coronal oblique shoulder MRI, with the angle formed by a line from the most lateral point of the inferior acromion to the inferior border of the glenoid and another line drawn along the superior and inferior most lateral glenoid (Fig. 1d).

Two independent well-trained orthopedic surgeons with experience with the shoulder performed the measurements while blinded to the patient’s diagnosis.

### Statistical analyses

All statistical analyses were performed using SPSS version 22 software. Interclass correlation coefficients (ICCs) were used to evaluate intraobserver and interobserver consistency in a two-way mixed model, and values greater than 0.75 were considered sufficient for reliability [22]. Associations were determined using the Pearson correlation coefficient (PCC). A PCC of less than 0.20 was determined to be poor; 0.21 to 0.40, fair; 0.41 to 0.60, moderate; 0.61 to 0.80, good; and 0.81 to 1.00, excellent [23]. The χ2 test was applied for categorical variables. The means for age, AHD, AI, LAA, and CSA from the two groups were compared using paired t tests. A *p* value less than 0.05 was considered significant. An area under the receiver operating characteristic (ROC) curve higher than 0.8 was considered significant. According to previous studies [9, 11, 24–26], AI has the minimum effect size(0.537) compared with other parameters, the sample size was calculated to be at least 74 each group with a power of 90 % and a *P* value of 0.05 using R statistical environment (v4.4.1) .

## Results

All demographic data of the patients are listed in Tables 1 and 2. In group 1, there were 31 men and 60 women with an average age of 60.8 ± 10.3, with 69.2 % right-handed. In group 2, there were 38 men and 53 women, with an average age of 59.4 ± 8.98 years and 59.3 % were right handed. There was no significant difference between the two groups in these basic demographic data.

**Table 1:** Characteristics of articular-sided partial thickness of rotator cuff tears and control group

	Group 1	Group 2	*P* value
N	91	91	
Age(years)	60.75 ± 10.3	59.42 ± 8.98	0.354
Gender			0.285
Male	31	38	
Female	60	53	
Limb			0.164
Left	28	37	
Right	63	54	
BMI(kg/m^2^)	24.68 ± 4.29	25.47 ± 3.91	0.193

Age and BMI are means ± standard division (SD)

*BMI* body mass index

**Table 2:** The relationship between variables and the articular-sided partial thickness of rotator cuff tears

	Group 1	Group 2	*P* value
Acromion type(n,%)			0.532
I	25(27.5 %)	34(37.4 %)	
II	48(45.0 %)	42(46.2 %)	
III	12(13.2 %)	9(9.90 %)	
IV	6(6.60 %)	6(6.60 %)	
CSA(°)	35.5 ± 4.07	33.7 ± 3.96	0.003
AI	0.683 ± 0.09	0.644 ± 0.08	0.002
LAA(°)	78.3 ± 4.97	79.5 ± 5.43	0.108
AHD(mm)	9.35 ± 1.29	9.13 ± 1.49	0.277

CSA, AI, LAA and AHD are means ± standard division (SD)

*CSA* critical shoulder angle, *AI* acromion index, *LAA* lateral acromial angle, *AHD* acromiohumeral distance

The ICCs of all imaging parameters were reliable, with intraobserver ICCs of 0.911 for AI, 0.979 for CSA, 0.972 for LAA and 0.934 for AHD, with interobserver ICCs of 0.837 for AI, 0.960 for CSA, 0.946 for LAA and 0.875 for AHD.

**Fig. 2:**
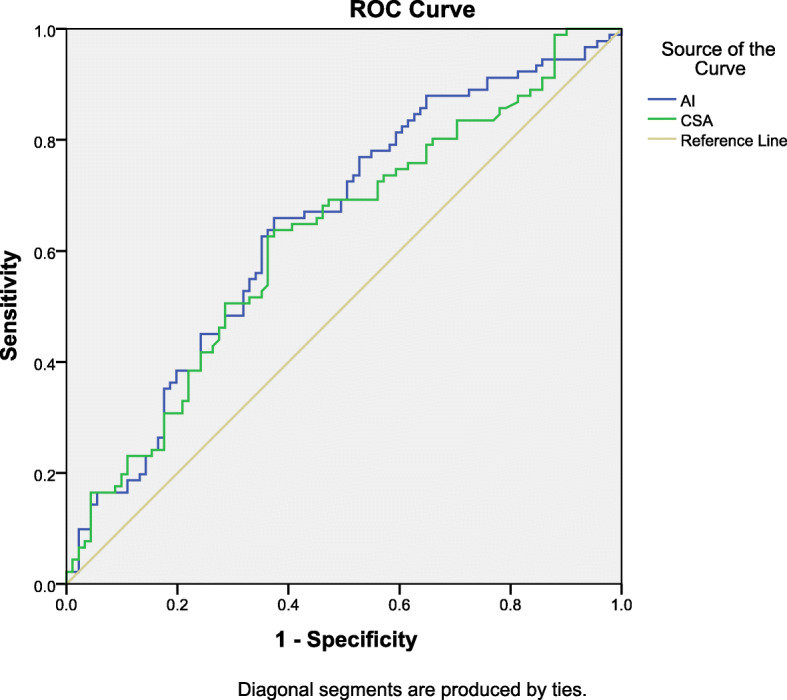
Receiver operating characteristic curves for the acromion index in blue and the critical shoulder angle in green. The reference line is indicated in yellow

The mean CSA values were significantly higher in articular-sided PTRCTs than in the controls (group 1: 35.5 ± 4.07 vs. group 2: 33.7 ± 3.96, *P* = 0.003). The mean AI was also significantly higher in articular-sided PTRCTs than in the controls (group 1: 0.683 ± 0.0857 vs. group 2: 0.644 ± 0.0804, *P* = 0.002). For CSA in the diagnosis of articular-sided PTRCTs, the area under the ROC curve was 0.624, and for AI in the diagnosis of articular-sided PTRCTs, the area under the ROC curve was 0.646 (Fig. 2).

Based on Bigliani [5] and Natsis’s [7] classifications, the most common acromion type was type II in both groups. In group 1, the acromions were 27.5 % type I, 45.0 % type II, 13.2 % type III and 6.60 % type IV. There were no differences within group 2, with 37.4 % type I, 46.2 % type II, 9.90 % type III and 6.60 % type IV.

The mean LAA values were not significantly different between group 1 and group 2 (78.3 ± 4.97 vs. 79.5 ± 5.43; *p* = 0.108). The average AHD was also similar between the two groups (group 1: 9.35 ± 1.29 vs. group 2: 9.13 ± 1.49, *P* = 0.277).

We found a good correlation between CSA and AI and a fair correlation between LAA and CSA and AI, AHD and CSA, and AHD and AI. Poor correlations were found between AHD and LAA and between age and acromion and the other parameters (Table 3).

**Table 3:** The correlations between variables

	AI	AHD	LAA	CSA	Age	Acromion type
AI						
Pearson Correlation	1	-0.207	-0.378	0.631	0.014	-0.075
Sig. (2-tailed)		0.005	0.000	0.000	0.854	0.317
AHD						
Pearson Correlation	-0.207	1	0.005	-0.232	0.098	0.083
Sig. (2-tailed)	0.005		0.943	0.002	0.189	0.264
LAA						
Pearson Correlation	-0.378	0.005	1	-0.357	-0.077	-0.007
Sig. (2-tailed)	0.000	0.943		0.000	0.299	0.929
CSA						
Pearson Correlation	0.631	-0.232	-0.357	1	0.123	-0.100
Sig. (2-tailed)	0.000	0.002	0.000		0.099	0.181
Age						
Pearson Correlation	0.014	0.098	-0.077	0.123	1	0.081
Sig. (2-tailed)	0.854	0.189	0.299	0.099		0.274
Acromion type						
Pearson Correlation	-0.075	0.083	-0.007	-0.100	0.081	1
Sig. (2-tailed)	0.317	0.264	0.929	0.181	0.274	

*AI* acromion index, *AHD* acromiohumeral distance, *LAA* lateral acromial angle, *CSA* critical shoulder angle

## Discussion

The main finding of the study was that statistically significant differences in AI and CSA were found between the articular-sided PTRCT group and the matched control group utilized MRI. Some studies have suggested that FTRCTs originate from partial tears [27]. Although the articular-sided PTRCTs is not in direct contact with the acromion, however, this study suggests a correlation between each other. It shows that the relationship between acromion and rotator cuff is more than direct attrition.

AI was first described by Nyffeler et al. [10] in 2006, who reported a larger AI in FTRCTs than in controls. In contrast to traumatic RCTs, Balke et al. [28] also showed a higher tendency in degenerative RCTs. While Pandey et al. [29] revealed an association between a higher AI and FTRCTs, they also showed no significant association in bursal-sided PTRCTs. Jung Ryul et al. [25] took articular-sided PTRCTs into consideration as a control group for FTRCTs and showed a significantly lower AI in articular-sided PTRCTs, the control group with intact rotator cuff tendons was not included. In our articular-side PTRCTs, with a mean value 0.04 higher than the control, the degree seems lower than the FTRCT with Nyffeler (0.09) and Pandey (0.06). This indicates that a higher degree difference in AI may cause a higher degree of RCT [25]. According to Thomas et al. [30], without distinguishing bursal-sided and articular-sided PTRCTs, they show a nonsignificant value between PTRCTs and FTRCTs. Therefore, a higher value of AI may exist in articular-sided PTRCTs and this was confirmed in our study. Although we can easily meet bursal-sided tear patients because bursal-sided tear had a higher degree of pain than articular-sided tear [31], but it is easier to make a tear on the articular side than on the bursal side [3]. Previous literature have shown that the articular side rotator cuff has poor vascularity [32], and biomechanical studies demonstrated the articular side elongated poorly, cause the ultimate stress is lower on the articular side [33], especially when there is a greater strain caused by the deltoid middle branch with a larger AI [10].

In recent years, CSA was first described by Moor et al. [13] as having a good association in FTRCTs with a higher value. As confirmed by later authors [24, 34], although Bjarnison et al. [35] reported no correlation between CSA and RCTs, there was a lack of distinguish PTRCTs and FTRCTs. Seo J et al. [36] found a higher CSA in articular-sided PTRCTs and no correlation with bursal-sided PTRCTs, and this result was consistent with another study described by Shinagawa et al. [34] with an overall lower CSA value in Asian people. By established shoulder model, Villatte et al [37] and Gerber et al [38] found that RCT overload particularly at low degrees of active abduction with a high CSA value. Because a higher CSA value may produce larger shear force, RCT need compensate additional shear force to maintain shoulder stability. This lead to increased muscle use may result in degenerative rotator cuff tears. The mean CSA value was different from that in our study, but we showed a similar tendency of higher CSA values in articular-sided PTRCTs.

Although a hook type seemed to have a higher association with RCTs according to Bigliani et al. [5], recently, an excessive hook type acromion with a slope larger than 43° was proven to be related to FTRCTs [23], but the overall acromion type showed no significance in RCT patients. By using shoulder magnetic resonance arthrography, Kim et al. [6] found that the acromion type was not associated with RCTs, bursal-sided PTRCTs or articular-sided PTRCTs. The association between rotator cuff tear and acromion type may be due to the wear of acromion to the rotator cuff on the bursal side. The acromion type had no correlation with articular-sided PTRCTs with an intact bursal side in this article, consistent with the previous studies. The mean value of LAA in our study was slightly lower in the patient group, but there was no statistical significance. In a previous study, Balke et al. [23] and Banas et al. [8] reported a significantly lower LAA value in degenerative RCTs. While the RCT group in Balke et al. did not differentiate the analysis by PTRCTs, bias caused by bursal-sided PTRCTs may exist. Saupe et al. [12] found that a shorter AHD was associated with FTRCT, and the progression of fatty degeneration increased migration. Goutallier et al. [11] proposed that an AHD less than 6 mm would be significantly associated with FTRCTs. In our study, an AHD similar to the control group and no AHD below 6 mm indicated that the humeral head did not migrate due to articular-sided PTRCTs. Previous study thought a smaller LAA may reduce the available volume and then impose pressure on the rotator cuff increase the tear risk. This is different from this study, while acromioplasty could increase the subacromial volume and AHD, if the main cause of articular-sided PTRCTs is not the acromion type, a smaller LAA and AHD, acromioplasty in surgical management would not be the optimum choice of treatment. Budoff et al. [39] reported PTRCTs needn’t acromioplasty, Weber [40] reported a higher rerupture rate and poor outcome despite adequate acromioplasty in articular-sided PTRCTs. This may indicated acromion type, a smaller LAA and AHD not the main cause of articular-sided PTRCTs.

A good correlation between CSA and AI was found in this article, which is similar to a previous study [41]. According to Moor et al. [13], CSA considered both glenoid and acromion anatomy as good parameters to predict RCT and osteoarthritis. Rhee et al. [42] showed good correlations between CSA and RCT and measurement of CSA did not calculate the morphology of the humeral head. At the same time, there are also literature reports that lateral acromial roof extension had a greater association with RCT than glenoid inclination [43]. AI is the ratio between lateral acromion with lateral humeral head to lateral glenoid [10]. In our study, glenohumeral arthritis was ruled out in all patients, so a good correlation appeared when the humeral head was normal. Although there was a higher CSA and AI in articular-sided PTRCTs, the area under the ROC curve showed a low diagnostic value; thus, the use of these parameters to diagnose articular-sided PTRCTs should proceed with caution.

Some limitations of this study should be considered. First, potential measurement bias may exist, although two well-trained orthopedists independently made the measurement without a previously known diagnosis. Second, this is a retrospective study in which all patients were symptomatic and underwent surgery, and selection bias may exist owing to asymptomatic articular-sided PTRCTs. Finally, since plain radiographs or computed tomography were not used, the accuracy of MRI measurement was not further verified, which also might have caused a bias.

## Conclusions

Our study revealed that higher AI and CSA were found in degenerative articular-sided PTRCTs. Acromial anatomy with a large acromial extension was associated with the occurrence of degenerative articular-sided PTRCTs.

## Data Availability

The datasets used and/or analysed during the current study are available from the corresponding author on reasonable request.

## Abbreviations

RCT: Rotator cuff tear
PTRCTs: Partial thickness of rotator cuff tears
FTRCT: Full thickness of rotator cuff tears
MRI: Magnetic resonance images
AHD: Acromiohumeral distance
LAA: Lateral acromial angle
AI: Acromion index
CSA: Critical shoulder angle
ICC: Interclass correlation coefficients
PCC: Pearson correlation coefficient
ROC: Receiver operating characteristic
SLAP: Superior labrum from anterior to posterior
BMI: Body mass index
